# A population‐based cohort of adult patients with diabetes mellitus in a Western District of Austria: The Diabetes Landeck cohort

**DOI:** 10.1002/edm2.395

**Published:** 2022-12-16

**Authors:** Veronika Haslwanter, Ursula Rochau, Uwe Siebert, Hans‐Robert Schönherr, Willi Oberaigner

**Affiliations:** ^1^ Department of Public Health, Health Services Research and Health Technology Assessment Institute of Public Health, Medical Decision Making and Health Technology Assessment, UMIT TIROL ‐ Private University for Health Sciences and Health Technology Hall in Tirol Austria; ^2^ Center for Health Decision Science, Departments of Epidemiology and Health Policy & Management Harvard T.H. Chan School of Public Health Boston Massachusetts USA; ^3^ Institute for Technology Assessment and Department of Radiology, Massachusetts General Hospital Harvard Medical School Boston Massachusetts USA; ^4^ Department of Internal Medicine St. Vinzenz Hospital Zams Zams Austria

**Keywords:** diabetes mellitus, gender, prevalence

## Abstract

**Introduction:**

Diabetes mellitus (DM) has become an important and exacerbating health epidemic, with severe consequences for both patients and health systems. The National Diabetes Strategy of Austria addresses the lack of high‐quality data on DM in Austria and the need for developing a national data network. The aims of our study are to establish a cohort including all adult diabetes patients in a district in western Austria, describe the demographic and clinical characteristics of this cohort, and provide an estimation of diabetes prevalence.

**Methods:**

We recruited a population‐based cohort of adult patients with a diagnosis of DM in cooperation with a network of all caregivers. Data collection was based on a case report form, including patient characteristics, clinical parameters and long‐term complications.

**Results:**

In total, 1845 patients with DM were recruited and analysed. We observed an overall prevalence of 5.3% [95% CI: 5.0%–5.5%]. For the subsequent main analysis, we included 1755 patients with DM after excluding 90 patients with gestational DM. There were significant differences between genders in the distribution of specific clinical parameters, patient characteristics, and the long‐term complications diabetic foot, amputation and cardiovascular disease.

**Conclusion:**

To the best of our knowledge, we established the first diabetes cohort study in Austria. Prevalence and the proportion of specific long‐term complications were lower when compared to the international context. We assume that the Diabetes Landeck Cohort has reached a high degree of completeness; however, we were not able to identify independent data sources for a valid check of completeness.

## Graphical abstract

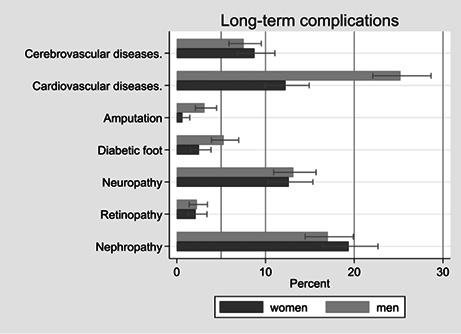

## 1 INTRODUCTION

Diabetes mellitus (DM) has become a considerable and exacerbating health epidemic that has severe consequences for both the patients and health systems.^1^ According to the tenth edition of the International Diabetes Federation (IDF) Atlas, approximately 537 million people aged 20 to 79 years suffered from DM worldwide in 2021, representing a prevalence of approximately 10.5%.^2^ The global prevalence is expected to increase in the future, with 783 million people predicted to suffer from DM by 2045, corresponding to a prevalence of around 10.9%.^2^ In Europe, one in eleven adults has DM, which means that 61 million people suffer from DM, and the prevalence is estimated to increase from 9.2% in 2021 to 10.4% in 2045. Furthermore, the economic burden associated with DM is substantial: The IDF reports that the share of the global health expenditure for DM ranges from 3.4% in the Middle East and North African region to 19.6% in Europe and to 43% in North America and the Caribbean.^2^ Patients with DM suffer from a lifelong burden caused by the treatment and disease complications. One of the main challenges is an increased risk of developing long‐term complications such as neuropathy, nephropathy and cardiovascular disease.^3^, ^4^ In addition, patients with DM have an increased risk of early mortality. Worldwide, about 6.7 million people aged between 20 and 79 years are estimated to die from DM or its complications in 2021.^2^

For Austria, the number of patients with DM aged 20–79 years in 2021 was estimated at about 450,000, corresponding to a prevalence of 6.6%. In addition, the number of undiagnosed DM cases was estimated at approximately 150,000.^2^ Due to the increasing number of patients with DM and the long‐term complications, considerable DM‐associated healthcare expenditures are also expected in Austria. The annual costs of DM and its comorbidities in Austria amount to an estimated 3 billion euros.^5^ Therefore, the main targets of the diabetes strategy of the Austrian Federal Ministry of Labor, Social Affairs, Health and Consumer Protection from 2017 are to reduce the incidence of DM and to prevent long‐term complications.^6^ This Austrian strategy document states that there is a lack and necessity of valid data on DM in Austria. Moreover, a report of the Austrian Court of Audition on DM prevention and DM care from 2019 points to the lack of high‐quality data.^7^ To provide valid data on DM in Austria, a project called the Diabetes Landeck Cohort was initiated, with the aim to set up a population‐based cohort of patients with DM in a district in western Austria. This cohort should serve as a basis to collect and analyse valid and comprehensive data on DM.

The aims of this project and study are to establish a cohort including all adult DM patients in a district in western Austria, describe the demographic and clinical characteristics of these patients, and provide an estimation of diabetes prevalence, both at an overall level and stratified by gender and age groups.

## 2 SUBJECTS AND METHODS

### 2.1 Settings of the study and data collection

The recruitment of the cohort titled ‘Diabetes Landeck Cohort’ started in 2018 and ended in June 2021. Eligible participants were adults (aged ≥20 years) with type 1 DM (T1DM) or type 2 DM (T2DM) or other types of diabetes (e.g. gestational, latent autoimmune diabetes in adults) and with the main residence in the district of Landeck in the western part of Austria. The district of Landeck is a well‐defined region with clear geographical borders, either high mountains within Austria or national borders towards Italy and Switzerland in the south. The district of Landeck with a population of 35,148 persons aged 20 and older consists of the city of Landeck with a population of 6120 persons aged 20 and older and 30 municipalities. This rural area is rather typical for rural areas in Austria. The study was conducted by the Research Unit of Diabetes Epidemiology of the Department of Public Health, Health Services Research and Health Technology Assessment at UMIT TIROL. Before beginning data collection, a case report form (CRF) was designed by diabetes and public health experts. The CRF includes questions on patient characteristics, and questions on time‐varying data based on visits at care units. A network connecting all care units has been established including 22 general practitioners, four diabetes specialists in private practice, the hospital in Zams, the Medical University Innsbruck and retirement homes in the district of Landeck. Data collection was mainly performed by two qualified study nurses who visited the care units. Direct documentation by the treating physician was also technically possible but conducted only in individual cases due to the heavy workload of the physicians caused by the COVID‐19 pandemic. All cohort data are stored in the pseudonymized web‐based database software ASKIMED.^8^ ASKIMED provides the possibility to store visits for each patient at different care units and allows the documentation by different persons and the mapping of the necessary access rights. A pseudonym was generated based on each patient's social security number (with a SHA‐2 procedure), which makes it impossible, due to the current computing performance, to identify the specific patient based on the pseudonym. For research purposes, data were transferred to the statistical software Stata (Version 17).^9^

In order to address aspects of data privacy, all patients had to sign a written consent form. For data protection reasons, the data of the very few patients who did not agree to sign the consent have not been included into the database for analysis. In addition, every care unit signed a contract stating the rights and obligations between the care units and the research group. The ethics committee at the Medical University of Innsbruck approved the complete project and the project was carried out in accordance with the Helsinki Declaration of 1975, as revised in 2008. A scientific advisory board supervised the study and decided in favour of a minimal data set to build up a population‐based cohort, given restrictions by the limited budget and the COVID‐19 pandemic.

### 2.2 Patient characteristics

We collected the following patient characteristics: diagnosis, age, migration background, diagnosis site, year of diagnosis, diabetes duration, family history of coronary heart disease and family history of DM, participation in the Austrian disease management program, smoking status, participation in a diabetes education program and sufficient knowledge on diabetes according to the physician.

All diagnoses were clinically confirmed according to the criteria of the Austrian Diabetes Society (ADS) based on a fasting glucose or oral glucose tolerance test or haemoglobin A1c (HbA1c).^10^ The study protocol did not include patients with prediabetes in the study population. Gestational diabetes (GDM) was documented separately. Diagnosis of GDM was based on the HAPO criteria.^11^ Age at the last visit was used for the analysis and was cut into categories, namely 20–49, 50–64, 65–74 and ≥75 years, taking into account the definition of geriatrics of the elderly/old person. The migration background was classified based on Schenk's approach and adapted to the Austrian situation.^12^ During documentation, participants were asked whether their DM diagnosis had been made in the hospital or at a physician's office (diagnosis site). For most patients, the year of diagnosis was recalled from patients' memory, and we decided to collect the exact year only for patients diagnosed during the past 10 years. The diabetes duration was defined as the difference between the year of DM diagnosis and the year of last contact with a care unit and was analysed in the following groups: 0–4 years, 5–9 years, and 10 years or longer. We documented family history of coronary heart disease and family history of DM. Family included children, parents and/or siblings. Participation in the Austrian Disease Management Program during the study period^13^ was also documented. Additionally, the smoking status at the time of diagnosis was recorded retrospectively in the categories ‘active smoker’ or ‘ex‐smoker’ and ‘never smoker’. We assessed whether each patient had participated at least once in a diabetes education program and whether the patient had sufficient knowledge on DM according to the physician.

Each patient's life status was verified by a study nurse who inspected hospital/private care records and local newspapers in case there were no up‐to‐date visit records. Pseudonymization prevented linking records with official mortality data. It was therefore impossible to systematically check the patients' life status.

### 2.3 Clinical parameters

Data collection included the following clinical parameters: body mass index (BMI), HbA1c, low‐density lipoprotein (LDL), systolic and diastolic blood pressure, microalbumin, physical activity, eye and foot inspection, hypoglycaemia (requiring external help for recovery), diabetes‐specific therapy and lipid therapy.

Mean values were computed for BMI, HbA1c, LDL, and systolic and diastolic blood pressure, which were collected at each visit. BMI was calculated according to the formula BMI = weight/height^2^ (kg/m^2^), and the classification was based on WHO recommendations. In addition, obesity was defined as a BMI ≥30 kg/m^2^.^14^ The mean values of HbA1c were categorized into four groups (<6.5%, 6.5%–7.49%, 7.5%–8.99% and >9%) based on the ADS guidelines.^10^ Increased blood pressure was defined as systolic pressure ≥140 mmHg or diastolic pressure ≥90 mmHg according to WHO guidelines.^15^ It should be noted that increased blood pressure is based on blood pressure measurements only. We did not collect information on the diagnosis of hypertension and/or medication.

LDL is the primary therapeutic target for lipid control in patients with DM. The LDL classification was based on current recommendations of the ESC/EAS.^16^ We did not collect information on the diagnosis of hyperlipidaemia, but we surveyed the proportion of patients with well‐controlled LDL levels. We recorded whether microalbumin was determined at least at one visit. Patients were asked if they were physically active (defined as at least moderate activity for less than two and a half hours per week). We also documented if an ophthalmologist had performed at least one eye inspection during visits. The inspections followed the recommendations of the ADS.^10^ Furthermore, we recorded foot inspections during the study period. Foot inspection was defined as at least the removal of shoes and socks and the examination of the feet by the diabetes assistant or physician. Furthermore, we noted severe hypoglycaemia (requiring external help for recovery) during the study period and calculated a cumulative number of events. Finally, we recorded information on the diabetes‐specific therapy. DM treatment categories were lifestyle adaptation only, metformin only, oral antidiabetic drugs (OADs) without metformin, insulin only, insulin and oral antidiabetic drugs, or another form of therapy. Each form of therapy was documented if it was observed during at least one visit over the study period.

### 2.4 Long‐term complications

We documented the following long‐term complications: neuropathy, nephropathy, retinopathy, cardiovascular and cerebrovascular disease, diabetic foot ulcers and amputations based on the recommendations of the ADS.^10^

Neuropathy is defined as nerve injuries due to DM and is confirmed with a positive monofilament test. Nephropathy requires positive albumin results at two subsequent visits. Retinopathy is diagnosed according to the guidelines provided by the Austrian Ophthalmologist Society.^17^ Cardiovascular late complication was defined as myocardial infarction, bypass or percutaneous coronary intervention. Cerebrovascular late complication was defined as minor and major strokes, including transient ischaemic attacks. We used the strict definition of the long‐term complication diabetic foot according to the guidelines of the ADS^10^ and therefore only collected information on the presence of diabetic foot ulcers. For amputations, we documented any non‐traumatic amputation due to diabetic foot ulcers. For all late complications, the year of the first occurrence was recorded if the diagnosis was made within the past 10 years. In all other cases, we only documented the diagnosis of the respective late complication without the year of occurrence. For the analysis we counted every long‐term complication, not only long‐term complications diagnosed in the study period.

### 2.5 Statistical analysis

Patient characteristics are described as counts and percentages for categorical data. We present all results stratified by gender and age groups (Tables S1–S2 only). Fisher's exact test or the chi‐squared test were used to test differences across gender and age groups. Statistical significance was established as *p* < .05. Cases with GDM that did not result in a life‐long type of DM (called ‘GDM only’) were described in a separate part because they differ in many clinical aspects. The main analysis of demographic and clinical parameters does not include cases with ‘GDM only’. In case of missing data, we report data and percentages for non‐missing values in a first step and present the number of cases with missing values in a second step; this procedure was adopted for every variable reported in the result tables.

For the computation of prevalence figures, we included all patients with DM detected in our study in the region of Landeck. Population data for the region of Landeck per age group were obtained from Statistics Austria.^18^ According to this population data, 35,148 individuals aged 20 and older were living in the district of Landeck at the time of the study, with slightly more women (17,839, 50.8%) than men (17,309, 49.2%). The population remained rather stable during the study period. Prevalence was computed for the entire district and for subregions, which are defined by the geographic structure of the following districts: Kaunertal, Stanzertal, Sonnenterasse, Oberes Gericht and Paznauntal. Prevalence and 95% confidence intervals (95% CI) are provided for the overall cohort and also stratified by gender, subregions and age groups. We applied the concept of period prevalence, that is, we included patients with at least one visit at a care unit stay during the study period as a prevalent case.

All statistical analyses were performed using Stata Version 17.^9^

## 3 RESULTS

### 3.1 Study population/diabetes Landeck cohort

The recruitment started in 2018 and ended in June 2021. In total, we recruited and analysed 1845 cases with DM including 90 cases with ‘GDM only’. For the main analysis, we excluded ‘GDM only’ cases resulting in a total 1755 cases with DM, 5.7% with T1DM, 92.4% with T2DM and 1.9% with another DM diagnosis. Of the 1755 patients, 25 (1.4%) died during the study and two subjects were lost in the follow‐up. At the last visit, 9.6% were aged 20–49 years, 27.2% were 50–64 years old, 26.6% were aged 65–74 years, and 36.6% were 75 years or older. We observed 812 (46.3%) women and 943 (53.7%) men. Patient characteristics of the cohort according to gender are presented in Table 1. We only describe results with significant differences between female and male patients. Age distributions differed between genders. For example, in the age group ≥75, the proportion of women with DM was higher than for men. Among patients with DM, women had a longer diabetes duration (55.9% ≥10 years vs. 49.2% in men) and more often reported a family history of diabetes (38% in women vs. 31.7% in men). In men, we observed a substantially higher percentage of active smokers (14.1% in men vs. 7.2% in women) and ex‐smokers compared to women (53.4% in men vs. 19.4% in women).

**TABLE 1:** Patient characteristics stratified by gender (*N* = 1755, ‘GDM only’ excluded)

	Female		Male		Total		*p*‐value^a^
	*N*	%^b^	*N*	%^b^	*N*	%^b^	
Age group							
20–49	67	8.3	101	10.7	168	9.6	<.001*
50–64	170	20.9	308	32.7	478	27.2	
65–74	215	26.5	251	26.6	466	26.6	
≥75	360	44.3	283	30.0	643	36.6	
Total	812	100.0	943	100.0	1755	100.0	
Missing values^c^	0	0	0	0	0	0	
Diagnosis							
T1DM	40	4.9	59	6.3	99	5.7	.112
T2DM	760	93.7	856	91.3	1616	92.4	
Other DM	11	1.4	23	2.5	34	1.9	
Total	811	100.0	938	100.0	1749	100.0	
Missing values	1	0.1	5	0.5	6	0.3	
Diagnosis site							
Hospital	243	32.1	325	36.8	568	34.6	.048*
Private practice	514	67.9	559	63.2	1073	65.4	
Total	757	100.0	884	100.0	1641	100.0	
Missing values	55	6.8	59	6.3	114	6.5	
Diabetes duration							
0–4 years	185	22.8	258	27.4	443	25.2	.016*
5–9 years	173	21.3	221	23.4	394	22.5	
≥10 years	454	55.9	464	49.2	918	52.3	
Total	812	100.0	943	100.0	1755	100.0	
Missing values	0	0	0	0	0	0	
Family history of diabetes							
No	482	62.0	611	68.3	1093	65.4	.007*
Yes	295	38.0	283	31.7	578	34.6	
Total	777	100.0	894	100.0	1671	100.0	
Missing values	35	4.3	49	5.2	84	4.8	
Family history of coronary heart disease							
No	621	79.7	727	81.4	1348	80.6	.386
Yes	158	20.3	166	18.6	324	19.4	
Total	779	100.0	893	100.0	1672	100.0	
Missing values	33	4.1	50	5.3	83	4.7	
Participation in disease management program							
No	726	89.4	843	89.4	1569	89.4	1.000
Yes	86	10.6	100	10.6	186	10.6	
Total	812	100.0	943	100.0	1755	100.0	
Missing values	0	0	0	0	0	0	
Life status							
Alive	800	98.5	928	98.4	1728	98.5	.416
Deceased	12	1.5	13	1.4	25	1.4	
Lost/moved	0	0.0	2	0.2	2	0.1	
Total	812	100.0	943	100.0	1755	100.0	
Missing values	0	0	0	0	0	0	
Migration background							
No	705	87.3	793	84.3	1498	85.6	.087
Yes	103	12.7	148	15.7	251	14.4	
Total	808	100.0	941	100.0	1749	100.0	
Missing values	4	0.5	2	0.2	6	0.3	
Smoking status							
Active smoker	55	7.2	125	14.1	180	10.9	.000*
Ex‐smoker	147	19.4	474	53.4	621	37.7	
Never smoker	557	73.4	288	32.5	845	51.3	
Total	759	100.0	887	100.0	1646	100.0	
Missing values	53	6.5	56	5.9	109	6.2	
Participation in education program							
No	168	21.0	209	22.5	377	21.8	.483
Yes	632	79.0	720	77.5	1352	78.2	
Total	800	100.0	929	100.0	1729	100.0	
Missing values	12	1.5	14	1.5	26	1.5	
Sufficient diabetes knowledge (according to physician)							
No	115	15.1	109	12.2	224	13.5	.097
Yes	648	84.9	785	87.8	1433	86.5	
Total	763	100.0	894	100.0	1657	100.0	
Missing values	49	6.0	49	5.2	98	5.6	

^a^ Fisher's exact test or chi‐squared test (non‐missing values only).

^b^ All percentages are based on non‐missing values (valid percentage).

^c^ Missing values are shown for each variable.

^*^ Statistical significance, *p* < .05.

Gender differences were found for specific clinical parameters, such as BMI, HbA1c, LDL, determination of microalbumin, DM therapy and physical activity. Table 2 shows the comparison of these characteristics between genders. We observed more overweight subjects (i.e. BMI = 25.0–29.99) in men than women. The proportion of obesity (i.e. BMI≥30) was similar for both genders. Women had a significantly greater proportion of well‐controlled HbA1c levels (HbA1c < 6.5) than men. The distribution of LDL showed a shift to higher values in men compared to women. Microalbumin was identified more frequently in men than in women, and more men were physically active (28.5% vs. 21.9%). We also observed differences in DM therapy. More women than men only adapted their lifestyle (22.3% vs. 16.2%). In men, metformin was more frequently used than in women, for example, more men (22.9%) received oral antidiabetic drugs therapy AND insulin than women (18.5%). Further details on clinical parameters are shown in Table 2.

**TABLE 2:** Clinical parameters stratified by gender (*N* = 1755, ‘GDM only’ excluded)

	Female		Male		Total		*p*‐value^a^
	*N*	%^b^	*N*	%^b^	*N*	%^b^	
BMI							
<18.5	9	1.1	5	0.5	14	0.8	.015*
18.5–24.99	174	22.0	165	18.0	339	19.8	
25.0–29.99	275	34.7	379	41.4	654	38.3	
≥30	334	42.2	367	40.1	701	41.0	
Total	792	100.0	916	100.0	1708	100.0	
Missing values^c^	20	2.5	27	2.9	47	2.7	
HbA1c							
0–6.49	320	41.9	325	36.2	645	38.8	.033*
6.5–7.49	254	33.3	303	33.7	557	33.5	
7.5–8.99	158	20.7	235	26.2	393	23.7	
9–99	31	4.1	35	3.9	66	4.0	
Total	763	100.0	898	100.0	1661	100.0	
Missing values	49	6.0	45	4.8	94	5.4	
LDL							
<55	77	10.2	110	12.6	187	11.5	.017*
55–69	92	12.2	111	12.7	203	12.5	
70–99	202	26.7	273	31.3	475	29.2	
≥100	386	51.0	378	43.3	764	46.9	
Total	757	100.0	872	100.0	1629	100.0	
Missing values	55	6.8	71	7.5	126	7.2	
Blood pressure (measured)							
Within normal range	585	74.0	670	73.0	1255	73.4	.661
Increased (≥140/90)	206	26.0	248	27.0	454	26.6	
Total	791	100.0	918	100.0	1709	100.0	
Missing values	21	2.6	25	2.7	46	2.6	
Microalbumin							
No	361	47.8	357	40.7	718	43.9	.004*
Yes	395	52.2	521	59.3	916	56.1	
Total	756	100.0	878	100.0	1634	100.0	
Missing values	56	6.9	65	6.9	121	6.9	
Diabetes therapy							
Only metformin	313	38.9	403	43.1	716	41.1	.008*
OAD without metformin	51	6.3	50	5.3	101	5.8	
Only insulin	106	13.2	112	12.0	218	12.5	
OAD + insulin	149	18.5	214	22.9	363	20.9	
Another form of therapy	6	0.7	5	0.5	11	0.6	
Only lifestyle adaptation	179	22.3	152	16.2	331	19.0	
Total	804	100.0	936	100.0	1740	100.0	
Missing values	8	1.0	7	0.7	15	0.9	
Number of occurrences of hypoglycaemia							
0	790	97.5	914	97.0	1704	97.3	.812
1	12	1.5	17	1.8	29	1.7	
≥2	8	1.0	11	1.2	19	1.1	
Total	810	100.0	942	100.0	1752	100.0	
Missing values	2	0.2	1	0.1	3	0.2	
Physical activity							
Inactive	568	78.1	609	71.5	1177	74.5	.003*
Active	159	21.9	243	28.5	402	25.5	
Total	727	100.0	852	100.0	1579	100.0	
Missing values	85	10.5	91	9.7	176	10.0	
Eye inspection							
No	303	37.4	355	37.7	658	37.6	.921
Yes	507	62.6	587	62.3	1094	62.4	
Total	810	100.0	942	100.0	1752	100.0	
Missing values	2	0.2	1	0.1	3	0.2	
Foot inspection							
No	170	21.0	185	19.6	355	20.3	.512
Yes	640	79.0	757	80.4	1397	79.7	
Total	810	100.0	942	100.0	1752	100.0	
Missing values	2	0.2	1	0.1	3	0.2	

Abbreviation: OAD, oral antidiabetic drug.

^a^ Fisher's exact test or chi‐squared test (non‐missing values only).

^b^ All percentages are based on non‐missing values (valid percentage).

^c^ Missing values are shown for each variable.

^*^ Statistical significance, *p* < .05.

#### 3.1.1 Long‐term complications

**FIGURE 1:**
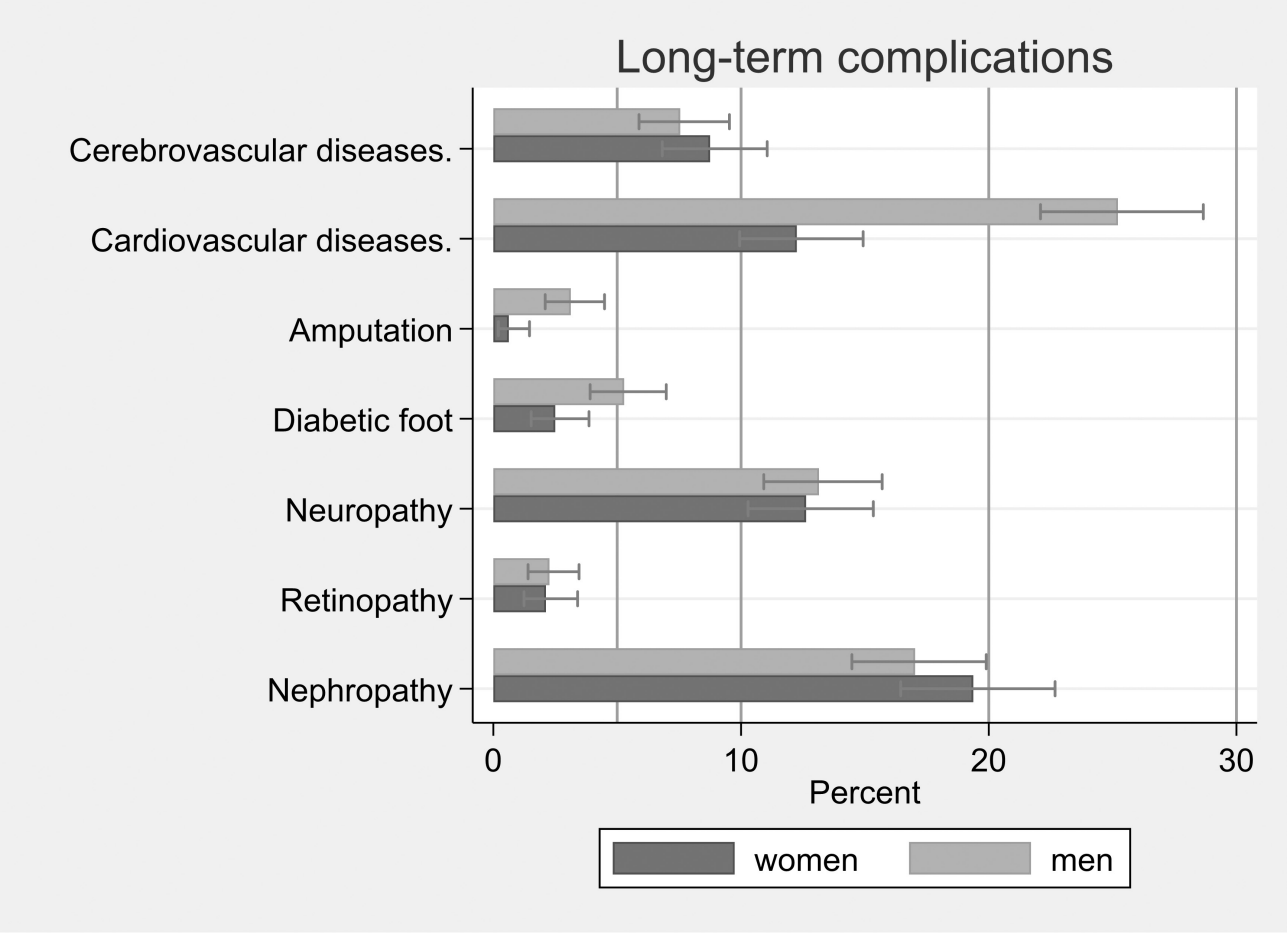
Long‐term complications stratified by gender (*N* = 1728, ‘GDM only’ and missing values excluded)

The most frequent long‐term complication was cardiovascular disease (*N* = 332, 19.2%), followed by nephropathy (*N* = 313, 18.1%) and neuropathy (*N* = 223, 12.9%). We observed significant differences between women and men for diabetic foot, amputation and cardiovascular disease. More men than women suffered from these three long‐term complications (Figure 1). Further details for the total population and genders are presented in Table 3.

**TABLE 3:** Long‐term complications stratified by gender (*N* = 1728, ‘GDM only’ and missing values excluded)

	Female		Male		Total		*p*‐value^a^
	*N*	%	*N*	%	*N*	%	
Nephropathy	155	19.4	158	17.0	313	18.1	.211
Retinopathy	17	2.1	21	2.3	38	2.2	.871
Neuropathy	101	12.6	122	13.1	223	12.9	.774
Diabetic foot	20	2.5	49	5.3	69	4.0	.003*
Amputation	5	0.6	29	3.1	34	2.0	<.001*
Cardiovascular disease	98	12.3	234	25.2	332	19.2	<.001*
Cerebrovascular disease^b^	70	8.8	70	7.5	140	8.1	.377

^a^ Fisher's exact test or chi‐squared test (non‐missing values only); total missing for long‐term complications: *N* = 27, 1.5%; women *N* = 12, 1.5% and men *N* = 15, 1.6%; multiple responses were possible.

^b^ Cerebrovascular late complication was defined as minor and major strokes, including transient ischaemic attacks.

^*^ Statistical significance, *p* < .05.

In our study, we observed 90 cases with GDM that did not develop T2DM. A mean value of HbA1c < 6 was documented for 94% of patients with GDM. Only three patients with GDM showed a HbA1c ≥ 6.5. Lifestyle adaptation was sufficient in 82.5% of patients with GDM, and insulin therapy was necessary for 13.4%.

See Tables S1–S2 for patient characteristics and clinical parameters according to the age groups 20–49, 50–64, 65–74 and ≥75 years. Briefly, we observed differences in the following patient characteristics: diabetes duration, migration background and smoking status. We also found a difference in the participation in education programmes and sufficient diabetes knowledge, but the percentage was still very high across all age groups. For the clinical parameters stratified by age group, we observed differences in the BMI, LDL, microalbumin, diabetes therapy, physical activity, foot inspection, and for most of the long‐term complications. Further details on patient characteristics and clinical parameters are described in Tables S1–S2.

### 3.2 Prevalence

In the age group ≥20 with a total population of 35,148, we observed an overall diabetes prevalence of 5.3% (95% CI: 5.0–5.5). Prevalence was slightly higher in men (5.5%, 95% CI: 5.1–5.8) than in women (5.1%, 95% CI: 4.7–5.4, difference not statistically significant). Prevalence differed between subregions: The lowest prevalence was observed in the subregion Sonnenterasse with 3.1% (95% CI: 2.4–3.9) and the highest in the central area Landeck and surroundings with 6.1% (95% CI: 5.7–6.5). The prevalence was 3.4% (95% CI: 2.9–3.9) in the region Paznauntal, 4.1% (95% CI: 3.1–5.5) in Kaunertal, 4.6% (95% CI: 4.1–5.3) in Stanzertal and 6.0% (95% CI: 5.4–6.6) in the region Oberes Gericht. Regarding gender, there were no statistically significant differences in prevalence for the subregions. The results for the subregions, genders and age groups are shown in Table 4.

**TABLE 4:** Prevalence by gender (*N* = 1845, with all DM, including ‘GDM only’)

	Females	Males	Total
	Prevalence in % (95% CI)	Prevalence in % (95% CI)	Prevalence in % (95% CI)
Total	5.1 (4.7–5.4)	5.5 (5.1–5.8)	5.3 (5.0–5.5)
Regions			
Landeck/surroundings	5.8 (5.3–6.4)	6.5 (5.9–7.1)	6.1 (5.7–6.5)
Oberes Gericht	5.9 (5.2–6.8)	6.0 (5.2–6.9)	6.0 (5.4–6.6)
Sonnenterasse	3.6 (2.4–4.9)	2.6 (1.7–3.7)	3.1 (2.4–3.9)
Kaunertal	4.6 (3.0–6.7)	3.7 (2.3–5.5)	4.1 (3.1–5.5)
Paznauntal	2.8 (2.2–3.6)	3.9 (3.2–4.8)	3.4 (2.9–3.9)
Stanzer Tal	4.4 (3.6–5.3)	4.9 (4.1–5.8)	4.6 (4.1–5.3)
Age groups			
20–49	1.8 (1.5–2.1)	1.1 (0.9–1.4)	1.5 (1.3–1.6)
50–64	3.6 (3.0–4.1)	6.3 (5.6–7.0)	4.9 (4.5–5.4)
65–74	11.0 (9.6–12.6)	14.3 (12.6–16.2)	12.5 (11.4–13.7)
≥75	15.4 (13.8–17.0)	18.1 (16.1–20.3)	16.5 (15.2–17.8)

Abbreviation: 95% CI, 95% confidence interval.

## 4 DISCUSSION

We established, to the best of our knowledge, the first population‐based cohort of patients with DM in Austria following the recommendations of the National Diabetes Strategy. Our cohort should support data‐driven healthcare decision‐making and should contribute improving outcomes of patients with DM.

In total, we analysed 1845 adult cases with DM (including ‘GDM only’) and observed an overall prevalence of 5.3%, with no statistically significant difference between genders but substantial differences between subregions. In addition, we identified differences between women and men in the following patient characteristics: diabetes duration, diagnosis site, family history of diabetes, smoking status, distribution of age groups, and for specific clinical parameters such as BMI, HbA1c, LDL, determination of microalbumin, DM therapy and physical activity. We also observed significant differences between female and male patients for the long‐term complications diabetic foot, amputation and cardiovascular disease.

In order to derive unmet needs and healthcare services quality gaps, it is important to set our results into the context of other countries. The prevalence of DM varies strongly across the globe and between European countries. The IDF Diabetes Atlas reports a prevalence ranging from 4.0% in Ireland to 15.9% in Turkey, and reports a prevalence for Austria of 6.6%, which is clearly below the European average of 9.2%.^2^ The overall prevalence of 5.3% in the district of Landeck is even lower than the prevalence estimate of the IDF for Austria, which lies above the 95% CI of the district Landeck (95% CI: 5.0%–5.5%). One reason for this difference within Austria could be the so‐called east–west gap in Austria. A report by the Federal Ministry of Health showed that mortality due to DM in western Austria is lower than in eastern Austria.^19^ There is also an evident east–west variation in cardiovascular mortality among persons over 64. Overweight/obesity, which is a major risk factor for DM,^20^ is an even more significant problem in the population over the age of 64 in the east of Austria than in the west.^19^ However, these estimates are not standardized, for example, for age, sociodemographic characteristics or proportion living in urban areas.

Our prevalence estimation for the district of Landeck is in line with the reported prevalence of 4% in Switzerland,^21^ which borders Landeck. Germany also shows a southwest‐to‐northeast gradient. The regional standardized prevalence was highest in the east, with 12.0% (95% CI: 10.3%–13.7%), and lowest in the south, with 5.8% (95% CI: 4.9%–6.7%).^22^ However, we cannot exclude an underestimation of the prevalence in our study because we were unable to validate the completeness of the population‐based cohort with independent data sources.

When comparing the frequencies with the literature, it should be noted that we included all DM patients treated in a region, not only those treated in a study using only a sample of the target population. Therefore, we predominantly compare our figures with results from population‐based diabetes registries. In addition, it is worth noting that our proportion of patients with high blood pressure should not be compared with hypertensive patients of study results from the published literature. The same is true for patients with higher LDL levels versus hyperlipidaemia.

In the following, we compare the parameters obesity, glycaemic control, smoking status, foot inspections and participation in a diabetes education program with data from the Scottish diabetes registry,^23^ the Swedish registry,^24^ and with results from the DAWN2 study.^25^ We also compare diabetes therapies with Austrian data^26^ and with data from a diabetes surveillance system for Germany at the Robert Koch Institute.^22^

In our study, 42.2% of women and 40.1% of men were obese. In Scotland, the proportion of obese patients was at 55%.^23^ In Sweden, obesity was also more frequent, with a prevalence of 61% for women and 54% for men, although data from Sweden are only available for patients in the age group 30–60.^24^ Overall, the proportion of obese patients in our study is lower compared to global numbers and the Austrian average,^27^ which fits the east–west gradient in Austria mentioned above.

Concerning glycaemic control, the proportion of patients with HbA1c < 7.5% was 55.4% in Scotland^23^ and 72.3% in our study (75.2% in women and 69.9% in men). One reason could be that the population in this western part of Austria is less obese and more physically active. Even overweight patients perform substantial physical activity. In addition, diabetic care is rather well structured.

The participation in a diabetes education program was 78.7% in the DAWN2 study,^25^ which is nearly identical to our results of 78.2%. The DAWN2 study reported 62.5% foot inspections (vs. 79.7% in the Diabetes Landeck Cohort); however, the DAWN2 data were based on self‐assessments. In the Diabetes Landeck Cohort, the proportion of active smokers was 10.9% (two times as many men than women), which is lower compared to 14.3% of the study of Panisch et al.^28^ and to 17.7% in Scotland.^23^

Our findings on diabetes therapy data do not describe the patients' current therapy (at the last contact) but rather summarize all therapy modalities that were documented during the study period. The proportion of patients with DM who did not need diabetes‐specific therapy (lifestyle adaptation only) is relatively high (19%) but is similar to the Swedish registry.^24^ The percentage of DM patients treated with OADs corresponds to 72% in the publication of Engler et al.,^26^ which describes patients in the diabetes register in Tyrol. Among 45‐ to 79‐year‐old people with T2DM in Germany, 29.6% of women and 37.2% of men received metformin monotherapy in 2010.^22^ This percentage is higher in our study (38.9% in women and 42.1% in men). Also, the percentage for OAD+ insulin is higher in our cohort, which included all patients with DM compared to the study across Germany that only assessed patients with T2DM.^22^ Comparisons of diabetes‐specific therapies with the literature should be carefully interpreted as many studies exclude elderly patients. In contrast, in our cohort, all patients with DM were registered, and the proportion of patients aged ≥75 was 36%.

When comparing our results with published data, it is essential to consider that the frequency of the respective long‐term complications depends on several factors, such as diabetes duration, age, gender and distribution of other risk factors. However, it also depends on the definition (and it is worthwhile to mention that definitions of each late complication differ in some respect), the extent of appropriate screening measures and the diagnostic methods used. Nonetheless, in the following paragraph, we attempt to compare our data on long‐term complications to the literature.

In Germany, the proportion of patients with DM with documented chronic kidney disease (as an indicator of nephropathy) was 15.1% (women: 14.9%; men: 15.3%) in 2013.^22^ For the Diabetes Landeck Cohort, the percentage is higher (18.1%) compared to Germany, but differences in the documentation and diagnosis standards complicate a comparison. Desphande et al.^29^ have provided an overview of the prevalence of the most common diabetes complications among individuals with T2M.^29^ They reported a frequency of nephropathy of 28%. As the disease DM progresses, the frequency of nephropathy is low in the first 10–15 years after the diagnosis and then increases significantly. In the Diabetes Landeck Cohort, the diabetes duration is less than 10 years in 50% of patients; this may explain the lower frequency of nephropathy, with 19.4% in women and 17.0% in men compared to the data of Desphande et al.^29^ Another explanation may be the relatively high proportion of patients with an HbA1c below 7.5%. The frequency of nephropathy correlates strongly with blood glucose control.^30^ In 2013, 13.5% of adults with DM had documented diabetic polyneuropathy in Germany (women: 12.7%; men: 14.4%).^22^ The proportion of patients with neuropathy is similar to that in our study (women: 12.6%; men: 13.1%). In Germany, 37.1% of adults with T2DM have cardiovascular disease, with a significantly lower prevalence in women (30.6%) than in men (42.8%), but this includes long‐term complications and comorbidities.^22^ In our study, we observed lower frequencies but also a significant gender difference (women: 12.3%; men: 25.2%) for cardiovascular disease as a long‐term complication. Our study and the meta‐analysis of Einarson^31^ show nearly identical results for cerebrovascular long‐term complications. The percentage of retinopathy in our cohort is very small (2.2%), but the prevalence of retinopathy increases progressively in patients with DM with increasing duration of the disease,^32^ and we could be confronted with problems in communication diagnoses from ophthalmologists to care givers. In Germany, 5.7% of women and 6.6% of men with DM had documented diabetic foot syndrome in 2013.^22^ For the Diabetes Landeck Cohort, the percentage is lower for women (2.5%) and similar for men (5.3%).

All patient data registered in this study were pseudonymized according to EU data protection laws. This means that the patient can no longer be identified in the research database, and implausible data can no longer be verified. The process of pseudonymization is based on the Austrian social security number, which means that patients cannot be registered without a social security number; however, the proportion of individuals without a social security number was fairly small in our study (about 0.3% of all patients).^33^

One strength of our study is that it covers a well‐defined population and region, all care providers are clearly identified, and the area is served almost exclusively by one hospital. We established a network of general practitioners, diabetes specialists in private practices, retirement homes and the hospital in a defined district. Cooperation with all care units was excellent, with very few exceptions. Another strength was the use of a workable documentation system. Furthermore, by limiting the data to a minimum basic dataset, we achieved an acceptable data quality and thus gained a valid representation of the quality of care. Our study design could be a prototype for other countries and can contribute to assess a good overview of the quality of care of patients with DM, given a limited budget. We were also able to identify the problems in the data collection, which is important for future updates of the cohort or planning of diabetes registries in Austria.

This study has several limitations. First, most of the study period was dominated by the COVID‐19 pandemic. The documentation took place during one of the most difficult periods in the Austrian healthcare system in recent decades, and this could have affected our data quality. The care units could not be visited at the predefined time intervals for a more extended period due to the COVID‐19 lockdown restrictions, and caregivers had less time to maintain and update records. This may lead to an underestimation of the true prevalence. Second, some general practitioners retired during the study, which limited cooperation. A third limitation that became evident during the study was the lack of coding DM diagnoses in most practice systems. This means that patients with DM can currently only be identified by a free text search, which is usually very time‐consuming and can be associated with measurement bias.^34^ This applies particularly to patients with DM who are not detected by the search criteria, which can lead to an underestimation of the true prevalence. It should also be considered that not all essential information is stored in the medical records and therefore was not accessible to the study nurse. Most physicians did not have the time to document or complete data themselves. Therefore, the majority of data documentation was completed by the study nurse who did not have direct contact with the patients. A fifth limitation of our study is that only a minimal data set was registered due to budget restrictions and the current conditions. For example, we were not able to collect data on triglycerides, ischaemic cardiomyopathy or other types of cardiomyopathy, or specific event types of cerebrovascular diseases or vascular events. As our results represent the population structure of the district Landeck and were not standardized to the Austrian population, crude comparisons with Austria should be interpreted with caution, see the discussion of the so‐called east–west gap in Austria. Another significant limitation, the need for a uniform definition of long‐term complications, applies to all published studies and was also a challenge we faced in our study. Finally, we were not able to verify the completeness of the prevalence estimate with independent data resources and could not systematically survey the patients' life statuses.

## 5 CONCLUSIONS

As explicitly stated in Austria's National Strategy Report on diabetes, there is a lack of high‐quality data on DM in the country.^6^ There are, to the best of our knowledge, no systematic population‐based data on patients with DM in Austria. The Diabetes Landeck Cohort closes this gap for one region in Austria and provides, for the first time in Austria, a nearly complete set of patients with DM living in a well‐defined region. Some results, such as the diabetes prevalence or the frequency of some long‐term complications, are lower compared to international data. We succeeded in establishing a population‐based cohort and related database; however, we were not able to identify independent sources to verify our results. Therefore, for the future, we strongly suggest evaluating both completeness and comparability of data with well‐accepted methods. In general, documentation by study nurses who should ideally be located in the care units is recommended to obtain valid data, because many important data are not stored in the practice systems or cannot be accessed in a systematic way. To access patients with specific diagnoses, support for the coding of diagnoses by physicians in private practices should be developed and applied in the practice systems. To make the best use of already existing data in the Austrian healthcare system, we recommend developing and/or optimizing systems to link different databases (e.g. civil registration and death data). The Diabetes Landeck Cohort should allow to evaluate and improve the quality of care of patients with DM in the future. In general, the cohort should be optimized and updated because high‐quality data provide an essential basis to optimize the care of patients with DM. The data could also be used to supplement a biobank, for long‐term monitoring of diabetes patients, for questions in health services research and healthcare economics, and for the investigation of new electronic communication methods between physicians and patients. Further research is needed, and in a subsequent step, we will extend this study by carefully taking the limitations into account.

## AUTHOR CONTRIBUTIONS

**Veronika Haslwanter:** Conceptualization (lead); data curation (equal); formal analysis (equal); investigation (equal); methodology (equal); project administration (equal); validation (equal); visualization (lead); writing – original draft (lead). **Ursula Rochau:** Supervision (supporting); writing – original draft (supporting); writing – review and editing (supporting). **Uwe Siebert:** Supervision (supporting); writing – original draft (supporting); writing – review and editing (supporting). **Hans Schoenherr:** Conceptualization (supporting); data curation (equal); methodology (equal). **Wilhelm Oberaigner:** Conceptualization (supporting); formal analysis (equal); methodology (equal); supervision (lead); writing – review and editing (equal).

## FUNDING INFORMATION

We thank the Tyrolean Health Fund (Tiroler Gesundheitsfond, TGF) for funding this project.

## CONFLICT OF INTEREST

The authors have no competing interests.

## ETHICS APPROVAL

The present study was approved by the Ethics Committee of the Medical University of Innsbruck and was carried out in accordance with the Helsinki Declaration of 1975, as revised in 2008.

## Supporting information


Tables S1‐S2

## Data Availability

The data that support the findings of this study are available from Tiroler Gesundheitsfond. Restrictions apply to the availability of these data, which were used under licence for this study.
