# Supplementary material for: A population‐based cohort of adult patients with diabetes mellitus in a Western District of Austria: The Diabetes Landeck cohort
Source: Endocrinol Diabetes Metab. 2022 Dec 16;6(2):e395. doi: 10.1002/edm2.395 (PMC10000636; doi:10.1002/edm2.395)
Supplement: Supplementary file 1 — Tables S1‐S2 [file EDM2-6-e395-s001.docx]

Supplementary material

***S 1: Patient characteristics stratified by age groups (N=1,755, “GDM only” excluded)***

|  | **20-49** | | **50-64** | | **65-74** | | **≥75** | | **Total** | | **p-value^a^** |
| --- | --- | --- | --- | --- | --- | --- | --- | --- | --- | --- | --- |
|  | N | % ^b^ | N | % ^b^ | N | % ^b^ | N | % ^b^ | N | % ^b^ |  |
| **Gender** |  |  |  |  |  |  |  |  |  |  |  |
| Female | 67 | 39.9 | 170 | 35.6 | 215 | 46.1 | 360 | 56.0 | 812 | 46.3 | <0.001* |
| Male | 101 | 60.1 | 308 | 64.4 | 251 | 53.9 | 283 | 44.0 | 943 | 53.7 |  |
| Total | 168 | 100.0 | 478 | 100.0 | 466 | 100.0 | 643 | 100.0 | 1,755 | 100.0 |  |
| **Diagnosis** |  |  |  |  |  |  |  |  |  |  |  |
| T1DM | 50 | 30.1 | 36 | 7.6 | 8 | 1.7 | 5 | 0.8 | 99 | 5.7 | <0.001* |
| T2DM | 103 | 62.0 | 431 | 90.5 | 446 | 95.7 | 630 | 98.3 | 1,610 | 92.1 |  |
| Other DM | 13 | 7.8 | 9 | 1.9 | 12 | 2.6 | 6 | 0.9 | 40 | 2.2 |  |
| Total | 166 | 100.0 | 476 | 100.0 | 466 | 100.0 | 641 | 100.0 | 1,749 | 100.0 |  |
| *Missing values* | *2* | *1.2* | *2* | *0.4* | *0* | *0.0* | *2* | *0.3* | *6* | *0.3* |  |
| **Diagnosis site** |  |  |  |  |  |  |  |  |  |  |  |
| Hospital | 59 | 35.1 | 148 | 31.0 | 141 | 30.3 | 220 | 34.2 | 568 | 32.4 | 0.377 |
| Private practice | 96 | 57.1 | 294 | 61.5 | 298 | 63.9 | 385 | 59.9 | 1,073 | 61.1 |  |
| Total | 155 | 100.0 | 442 | 100.0 | 439 | 100.0 | 605 | 100.0 | 1,641 | 100.0 |  |
| *Missing values* | *13* | *7.7* | *36* | *7.5* | *27* | *5.8* | *38* | *5.9* | *114* | *6.5* |  |
| **Duration diabetes** |  |  |  |  |  |  |  |  |  |  |  |
| 0–4 years | 81 | 48.2 | 165 | 34.5 | 103 | 22.1 | 94 | 14.6 | 443 | 25.2 | <0.001* |
| 5–9 years | 36 | 21.4 | 122 | 25.5 | 110 | 23.6 | 126 | 19.6 | 394 | 22.5 |  |
| ≥10 years | 51 | 30.4 | 191 | 40.0 | 253 | 54.3 | 423 | 65.8 | 918 | 52.3 |  |
| Total | 168 | 100.0 | 478 | 100.0 | 466 | 100.0 | 643 | 100.0 | 1,755 | 100.0 |  |
| *Missing values* | *0* | *0* | *0* | *0* | *0* | *0* | *0* | *0* | *0* | *0* |  |
| **Family history of diabetes** |  |  |  |  |  |  |  |  |  |  |  |
| No | 102 | 65.4 | 261 | 56.9 | 291 | 64.8 | 439 | 72.3 | 1,093 | 65.4 | <0.001* |
| Yes | 54 | 34.6 | 198 | 43.1 | 158 | 35.2 | 168 | 27.7 | 578 | 34.6 |  |
| total | 156 | 100.0 | 459 | 100.0 | 449 | 100.0 | 607 | 100.0 | 1,671 | 100.0 |  |
| *Missing values* | *12* | *7.1* | *19* | *4.0* | *17* | *3.6* | *36* | *5.6* | *84* | *4.8* |  |
| **Family history of coronary heart disease** |  |  |  |  |  |  |  |  |  |  |  |
| No | 137 | 87.8 | 347 | 75.6 | 363 | 80.8 | 501 | 82.4 | 1,348 | 80.6 | 0.003* |
| Yes | 19 | 12.2 | 112 | 24.4 | 86 | 19.2 | 107 | 17.6 | 324 | 19.4 |  |
| total | 156 | 100.0 | 459 | 100.0 | 449 | 100.0 | 608 | 100.0 | 1,672 | 100.0 |  |
| *Missing values* | *12* | *7.1* | *19* | *4.0* | *17* | *3.6* | *35* | *5.4* | *83* | *4.7* |  |
| **Participation disease management program** |  |  |  |  |  |  |  |  |  |  |  |
| No | 147 | 87.5 | 414 | 86.6 | 422 | 90.6 | 586 | 91.1 | 1,569 | 89.4 | 0.065 |
| Yes | 21 | 12.5 | 64 | 13.4 | 44 | 9.4 | 57 | 8.9 | 186 | 10.6 |  |
| Total | 168 | 100.0 | 478 | 100.0 | 466 | 100.0 | 643 | 100.0 | 1,755 | 100.0 |  |
| *Missing values* | *0* | *0* | *0* | *0* | *0* | *0* | *0* | *0* | *0* | *0* |  |
| **Life status** |  |  |  |  |  |  |  |  |  |  |  |
| Alive | 167 | 99.4 | 474 | 99.2 | 463 | 99.4 | 624 | 97.0 | 1,728 | 98.5 | <0.001* |
| Deceased | 1 | 0.6 | 2 | 0.4 | 3 | 0.6 | 19 | 3.0 | 25 | 1.4 |  |
| Lost/moved | 0 | 0.0 | 2 | 0.4 | 0 | 0.0 | 0 | 0.0 | 2 | 0.1 |  |
| Total | 168 | 100.0 | 478 | 100.0 | 466 | 100.0 | 643 | 100.0 | 1,755 | 100.0 |  |
| *Missing values* | *0* | *0* | *0* | *0* | *0* | *0* | *0* | *0* | *0* | *0* |  |
| **Migration background** |  |  |  |  |  |  |  |  |  |  |  |
| No | 134 | 80.2 | 365 | 77.0 | 397 | 85.4 | 602 | 93.6 | 1,498 | 85.6 | <0.001* |
| Yes | 33 | 19.8 | 109 | 23.0 | 68 | 14.6 | 41 | 6.4 | 251 | 14.4 |  |
| Total | 167 | 100.0 | 474 | 100.0 | 465 | 100.0 | 643 | 100.0 | 1,749 | 100.0 |  |
| *Missing values* | *1* | *0.6* | *4* | *0.8* | *1* | *0.2* | *0* | *0.0* | *6* | *0.3* |  |
| **Smoking status** |  |  |  |  |  |  |  |  |  |  |  |
| Active smoker | 36 | 23.7 | 86 | 19.1 | 40 | 9.2 | 18 | 2.9 | 180 | 10.9 | <0.001* |
| Ex-smoker | 33 | 21.7 | 173 | 38.4 | 203 | 46.9 | 212 | 34.7 | 621 | 37.7 |  |
| Never smoker | 83 | 54.6 | 191 | 42.4 | 190 | 43.9 | 381 | 62.4 | 845 | 51.3 |  |
| Total | 152 | 100.0 | 450 | 100.0 | 433 | 100.0 | 611 | 100.0 | 1,646 | 100.0 |  |
| *Missing values* | *16* | *9.5* | *28* | *5.9* | *33* | *7.1* | *32* | *5.0* | *109* | *6.2* |  |
| **Participation in education program** |  |  |  |  |  |  |  |  |  |  |  |
| No | 27 | 16.6 | 88 | 18.7 | 99 | 21.3 | 163 | 25.8 | 377 | 21.8 | 0.011* |
| Yes | 136 | 83.4 | 383 | 81.3 | 365 | 78.7 | 468 | 74.2 | 1,352 | 78.2 |  |
| Total | 163 | 100.0 | 471 | 100.0 | 464 | 100.0 | 631 | 100.0 | 1,729 | 100.0 |  |
| *Missing values* | *5* | *3.0* | *7* | *1.5* | *2* | *0.4* | *12* | *1.9* | *26* | *1.5* |  |
| **Sufficient diabetes knowledge** |  |  |  |  |  |  |  |  |  |  |  |
| No | 18 | 11.8 | 58 | 12.9 | 46 | 10.5 | 102 | 16.6 | 224 | 13.5 | 0.033* |
| Yes | 134 | 88.2 | 391 | 87.1 | 394 | 89.5 | 514 | 83.4 | 1,433 | 86.5 |  |
| Total | 152 | 100.0 | 449 | 100.0 | 440 | 100.0 | 616 | 100.0 | 1,657 | 100.0 |  |
| *Missing values* | *16* | *9.5* | *29* | *6.1* | *26* | *5.6* | *27* | *4.2* | *98* | *5.6* |  |

^a^ Fisher’s exact test or chi-squared test (non-missing values only), ^b^ all percentages are based on non-missing values (valid percentage), ^c^ missing values are shown for each variable.

***S 2: Clinical parameters stratified by age groups (N=1,755, “GDM only” excluded)***

|  | **20-49** | | **50-64** | | **65-74** | | **≥75** | | **Total** | | **p-value^a^** |
| --- | --- | --- | --- | --- | --- | --- | --- | --- | --- | --- | --- |
|  | N | % ^b^ | N | % ^b^ | N | % ^b^ | N | % ^b^ | N | % ^b^ |  |
| **BMI** |  |  |  |  |  |  |  |  |  |  |  |
| <18.5 | 4 | 2.5 | 2 | 0.4 | 4 | 0.9 | 4 | 0.6 | 14 | 0.8 | <0.001* |
| 18.5–24.99 | 43 | 26.5 | 62 | 13.3 | 79 | 17.4 | 155 | 24.8 | 339 | 19.8 |  |
| 25.0–29.99 | 42 | 25.9 | 172 | 36.8 | 168 | 37.0 | 272 | 43.5 | 654 | 38.3 |  |
| ≥30 | 73 | 45.1 | 231 | 49.5 | 203 | 44.7 | 194 | 31.0 | 701 | 41.0 |  |
| Total | 162 | 100.0 | 467 | 100.0 | 454 | 100.0 | 625 | 100.0 | 1,708 | 100.0 |  |
| *Missing values^c^* | *6* | *3.6* | *11* | *2.3* | *12* | *2.6* | *18* | *2.8* | *47* | *2.7* |  |
| **HbA1c** |  |  |  |  |  |  |  |  |  |  |  |
| 0–6.49 | 60 | 39.2 | 155 | 34.6 | 175 | 40.0 | 255 | 40.9 | 645 | 38.8 | 0.359 |
| 6.5–7.49 | 48 | 31.4 | 150 | 33.5 | 147 | 33.6 | 212 | 34.0 | 557 | 33.5 |  |
| 7.5–8.99 | 29 | 19.0 | 95 | 21.2 | 84 | 19.2 | 107 | 17.2 | 315 | 19.0 |  |
| 9–99 | 16 | 10.5 | 48 | 10.7 | 31 | 7.1 | 49 | 7.9 | 144 | 8.7 |  |
| Total | 153 | 100.0 | 448 | 100.0 | 437 | 100.0 | 623 | 100.0 | 1,661 | 100.0 |  |
| *Missing values* | *15* | *8.9* | *30* | *6.3* | *29* | *6.2* | *20* | *3.1* | *94* | *5.4* |  |
| **LDL** |  |  |  |  |  |  |  |  |  |  |  |
| <55 | 10 | 6.5 | 39 | 9.0 | 58 | 13.5 | 80 | 13.1 | 187 | 11.5 | <0.001* |
| 55–69 | 9 | 5.9 | 51 | 11.8 | 57 | 13.3 | 86 | 14.1 | 203 | 12.5 |  |
| 70–99 | 47 | 30.7 | 112 | 25.8 | 135 | 31.4 | 181 | 29.6 | 475 | 29.2 |  |
| ≥100 | 87 | 56.9 | 232 | 53.5 | 180 | 41.9 | 265 | 43.3 | 764 | 46.9 |  |
| Total | 153 | 100.0 | 434 | 100.0 | 430 | 100.0 | 612 | 100.0 | 1,629 | 100.0 |  |
| *Missing values* | *15* | *8.9* | *44* | *9.2* | *36* | *7.7* | *31* | *4.8* | *126* | *7.2* |  |
| **Microalbumin** |  |  |  |  |  |  |  |  |  |  |  |
| No | 37 | 24.7 | 168 | 38.3 | 183 | 42.3 | 330 | 53.9 | 718 | 43.9 | <0.001* |
| Yes | 113 | 75.3 | 271 | 61.7 | 250 | 57.7 | 282 | 46.1 | 916 | 56.1 |  |
| Total | 150 | 100.0 | 439 | 100.0 | 433 | 100.0 | 612 | 100.0 | 1,634 | 100.0 |  |
| *Missing values* | *18* | *10.7* | *39* | *8.2* | *33* | *7.1* | *31* | *4.8* | *121* | *6.9* |  |
| **Blood pressure (measured)** |  |  |  |  |  |  |  |  |  |  |  |
| Within normal range | 123 | 75.9 | 336 | 72.1 | 333 | 73.8 | 463 | 73.5 | 1,255 | 73.4 | 0.816 |
| Increased (≥140/90) | 39 | 24.1 | 130 | 27.9 | 118 | 26.2 | 167 | 26.5 | 454 | 26.6 |  |
| Total | 162 | 100.0 | 466 | 100.0 | 451 | 100.0 | 630 | 100.0 | 1,709 | 100.0 |  |
| *Missing values* | *6* | *3.6* | *12* | *2.5* | *15* | *3.2* | *13* | *2.0* | *46* | *2.6* |  |
| **Diabetes therapy** |  |  |  |  |  |  |  |  |  |  |  |
| Metformin alone | 46 | 27.9 | 219 | 46.0 | 217 | 47.0 | 234 | 36.7 | 716 | 41.1 | <0.001* |
| OAD^d^ without metformin | 8 | 4.8 | 17 | 3.6 | 16 | 3.5 | 60 | 9.4 | 101 | 5.8 |  |
| Only insulin | 52 | 31.5 | 48 | 10.1 | 37 | 8.0 | 81 | 12.7 | 218 | 12.5 |  |
| OAD^d^ + insulin | 22 | 13.3 | 109 | 22.9 | 106 | 22.9 | 126 | 19.8 | 363 | 20.9 |  |
| Another form of therapy | 4 | 2.4 | 4 | 0.8 | 1 | 0.2 | 2 | 0.3 | 11 | 0.6 |  |
| Only lifestyle adaptation | 33 | 20.0 | 79 | 16.6 | 85 | 18.4 | 134 | 21.0 | 331 | 19.0 |  |
| Total | 165 | 100.0 | 476 | 100.0 | 462 | 100.0 | 637 | 100.0 | 1,740 | 100.0 |  |
| *Missing values* | *3* | *1.8* | *2* | *0.4* | *4* | *0.9* | *6* | *0.9* | *15* | *0.9* |  |
| **Number of occurrences hypoglycemias** |  |  |  |  |  |  |  |  |  |  |  |
| 0 | 159 | 95.2 | 467 | 97.7 | 454 | 97.8 | 624 | 97.0 | 1,704 | 97.3 | 0.202 |
| 1 | 7 | 4.2 | 6 | 1.3 | 5 | 1.1 | 11 | 1.7 | 29 | 1.7 |  |
| ≥2 | 1 | 0.6 | 5 | 1.0 | 5 | 1.1 | 8 | 1.2 | 19 | 1.1 |  |
| Total | 167 | 100.0 | 478 | 100.0 | 464 | 100.0 | 643 | 100.0 | 1,752 | 100.0 |  |
| *Missing values* | *1* | *0.6* | *0* | *0.0* | *2* | *0.4* | *0* | *0.0* | *3* | *0.2* |  |
| **Physical activity** |  |  |  |  |  |  |  |  |  |  |  |
| Inactive | 81 | 55.5 | 309 | 73.0 | 292 | 70.0 | 495 | 83.5 | 1,177 | 74.5 | <0.001* |
| Active | 65 | 44.5 | 114 | 27.0 | 125 | 30.0 | 98 | 16.5 | 402 | 25.5 |  |
| Total | 146 | 100.0 | 423 | 100.0 | 417 | 100.0 | 593 | 100.0 | 1,579 | 100.0 |  |
| *Missing values* | *22* | *13.1* | *55* | *11.5* | *49* | *10.5* | *50* | *7.8* | *176* | *10.0* |  |
| **Eye inspection** |  |  |  |  |  |  |  |  |  |  |  |
| No | 68 | 40.7 | 196 | 41.0 | 166 | 35.8 | 228 | 35.5 | 658 | 37.6 | 0.174 |
| Yes | 99 | 59.3 | 282 | 59.0 | 298 | 64.2 | 415 | 64.5 | 1,094 | 62.4 |  |
| total | 167 | 100.0 | 478 | 100.0 | 464 | 100.0 | 643 | 100.0 | 1,752 | 100.0 |  |
| *Missing values* | *1* | *0.6* | *0* | *0.0* | *2* | *0.4* | *0* | *0.0* | *3* | *0.2* |  |
| **Foot inspection** |  |  |  |  |  |  |  |  |  |  |  |
| No | 47 | 28.1 | 109 | 22.8 | 104 | 22.4 | 95 | 14.8 | 355 | 20.3 | <0.001* |
| Yes | 120 | 71.9 | 369 | 77.2 | 360 | 77.6 | 548 | 85.2 | 1,397 | 79.7 |  |
| Total | 167 | 100.0 | 478 | 100.0 | 464 | 100.0 | 643 | 100.0 | 1,752 | 100.0 |  |
| *Missing values* | *1* | *0.6* | *0* | *0.0* | *2* | *0.4* | *0* | *0.0* | *3* | *0.2* |  |
|  |  |  |  |  |  |  |  |  |  |  |  |

^a^ Fisher’s exact test or chi-squared test (non-missing values only), ^b^ all percentages are based on non-missing values (valid percentage), ^c^ missing values are shown for each variable, ^d^ OAD: oral antidiabetic drug.
